# Structural characteristics of the bone surrounding dental implants placed into the tail-suspended mice

**DOI:** 10.1186/s40729-021-00374-3

**Published:** 2021-09-01

**Authors:** Yuto Otsu, Satoru Matsunaga, Takehiro Furukawa, Kei Kitamura, Masaaki Kasahara, Shinichi Abe, Takayoshi Nakano, Takuya Ishimoto, Yasutomo Yajima

**Affiliations:** 1 Department of Oral and Maxillofacial Implantology, Tokyo Dental College, 2-9-18 Kandamisaki-cho, Chiyoda-ku, Tokyo, 101-0061 Japan; 2 Oral Health Science Center, Tokyo Dental College, 2-9-18 Kandamisaki-cho, Chiyoda-ku, Tokyo, 101-0061 Japan; 3 Department of Anatomy, Tokyo Dental College, 2-9-18 Kandamisaki-cho, Chiyoda-ku, Tokyo, 101-0061 Japan; 4 Department of Histology and Developmental Biology, Tokyo Dental College, 2-9-18 Kandamisaki-cho, Chiyoda-ku, Tokyo, 101-0061 Japan; 5 Department of Dental Materials Science, Tokyo Dental College, 2-9-18 Kandamisaki-cho, Chiyoda-ku, Tokyo, 101-0061 Japan; 6 Division of Materials and Manufacturing Science, Graduate School of Engineering, Osaka University, 2-1, Yamada-oka, Suita, Osaka, 565-0871 Japan

**Keywords:** Tail suspension, Femur, Bone quality, Biological apatite orientation, Collagen fiber, Dental implant

## Abstract

**Background:**

There are many unclear points regarding local structural characteristics of the bone surrounding the implant reflecting the mechanical environment.

**Purpose:**

The purpose of this study is to quantitatively evaluate bone quality surrounding implants placed into the femurs of mice in an unloading model, and to determine the influence of the mechanical environment on bone quality.

**Methods:**

Twenty 12-week-old male C57BL6/NcL mice (*n* = 5/group) were used as experimental animals. The mice were divided into two groups: the experimental group (*n* = 10) which were reared by tail suspension, and the control group (*n* = 10) which were reared normally. An implant was placed into the femur of a tail-suspended mouse, and after the healing period, they were sacrificed and the femur was removed. After micro-CT imaging, Villanueva osteochrome bone stain was performed. It was embedded in unsaturated polyester resin. The polymerized block was sliced passing through the center of the implant body. Next, 100-μm-thick polished specimens were prepared with water-resistant abrasive paper. In addition to histological observation, morphometric evaluation of cancellous bone was performed, and the anisotropy of collagen fibers and biological apatite (BAp) crystals was analyzed.

**Results:**

As a result, the femoral cortical bone thickness and new peri-implant bone mass showed low values in the tail suspension group. The uniaxial preferential orientation of BAp *c*-axis in the femoral long axis direction in the non-implant groups, but biaxial preferential orientation of BAp *c*-axis along the long axis of implant and femoral long axis direction were confirmed in new bone reconstructed by implant placement. Collagen fiber running anisotropy and orientation of BAp *c*-axis in the bone surrounding the implant were not significantly different due to tail suspension.

**Conclusions:**

From the above results, it was clarified that bone formation occurs surrounding the implant even under extremely low load conditions, and bone microstructure and bone quality adapted to the new mechanical environment are acquired.

## Background

For successful implant treatment, osseointegration is essential [1, 2]. It was reported that the force applied to the implant is always optimized by bone remodeling to support and buffer the functional pressure [3, 4]. In particular, the harmful effects of overload on implants are widely known, and are considered to be one of the factors that cause bone resorption surrounding the implant and decrease bone density [5, 6]. Ogiso et al. [7] reported that the bone surrounding the implant is composed of dense lamellar bone, and its thickness increases as functional pressure continues. Regarding the load applied to implants, many researchers [8, 9] describe that adaptive loading increases bone-implant contact rate, and excessive loading causes bone resorption surrounding the implant. On the other hand, Vandamme et al. [10] reported that the immediate load after implant placement promotes bone formation around the implant, and the unloading period with no load is disadvantageous for obtaining osseointegration.

In addition, Naert et al. [11] reported that bone resorption does not occur even when overload is added to the implant unless there is a bacterial infection surrounding the implant. On the other hand, Isidor [12] reported that occlusal overloading can result in a complete or partial loss of osseointegration at the histological level. However, the existence of complex factors such as bacteria and forces or their interactions makes it difficult to verify the relationship between the bone surrounding the implant and load [13].

On the other hand, we noted that the femur can be released from mechanical load by suspending the tail of the mouse [14], and the femur in this model has the advantage of infinitely less exposure to load. By using this model, we thought that it is possible to quantitatively evaluate bone structural characteristics surrounding an implant when the implant is placed in the unloaded bone.

In previous studies of the bone-implant interface, Romanos et al. [15] mainly evaluated only whole bone mass, so there are many unclear points regarding local structural characteristics of the bone surrounding the implant reflecting the mechanical environment. According to recommendations on osteoporosis by National Institutes of Health [16] in 2000, bone strength is considered to be insufficient by evaluating only bone mass, and it is considered that bone quality factors, such as bone microstructure, bone turnover, degree of calcification, and accumulation of microdamage, control bone mechanical function. Nakano et al. [17], by using a material engineering method, reported that the bone quality strongly reflect the local load environment. By applying this method, it is possible to accurately predict the mechanical function of the bone surrounding the implant.

Therefore, in this study, we performed a histological examination of the peri-implant bone in the femur of a tail-suspended mouse, and analyzed the anisotropy of collagen fibers and biological apatite (BAp) crystals. The purpose of this study is to quantitatively evaluate bone quality surrounding the implants placed into the femurs of mice in an unloading model, and to determine the influence of the mechanical environment on bone quality.

## Methods

### Animals

**Fig. 1:**
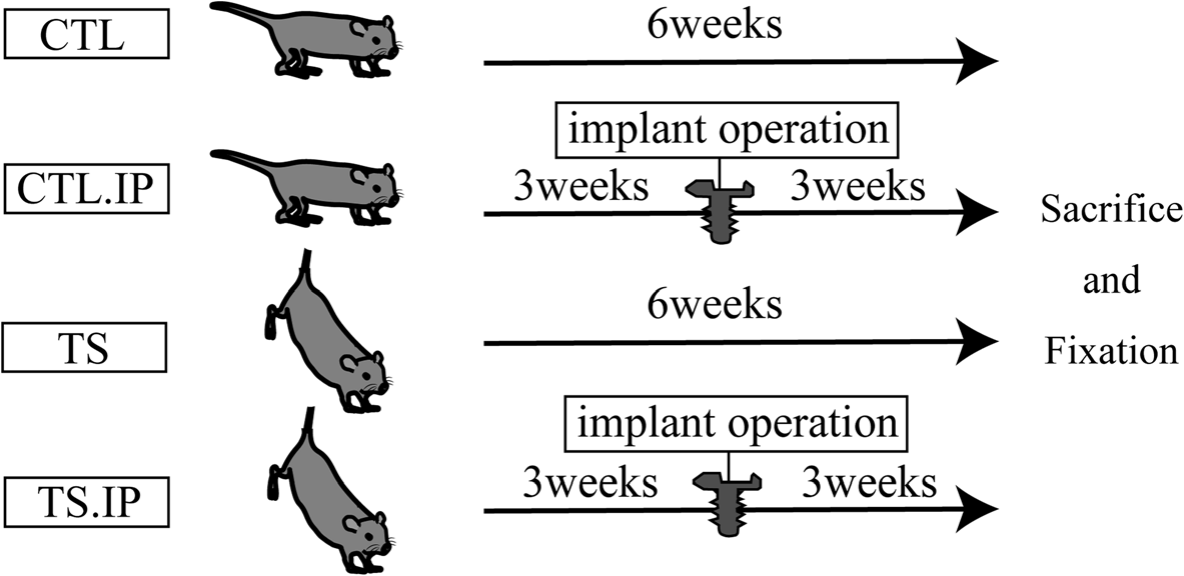
Experimental protocol. We compared tail suspension mice to normal-reared mice. Implant surgery was performed after 3 weeks of tail suspension. *CTL* control, *CTL*.*IP* control with implant, *TS* tail suspension, *TS*.*IP* tail suspension with implant

**Fig. 2:**
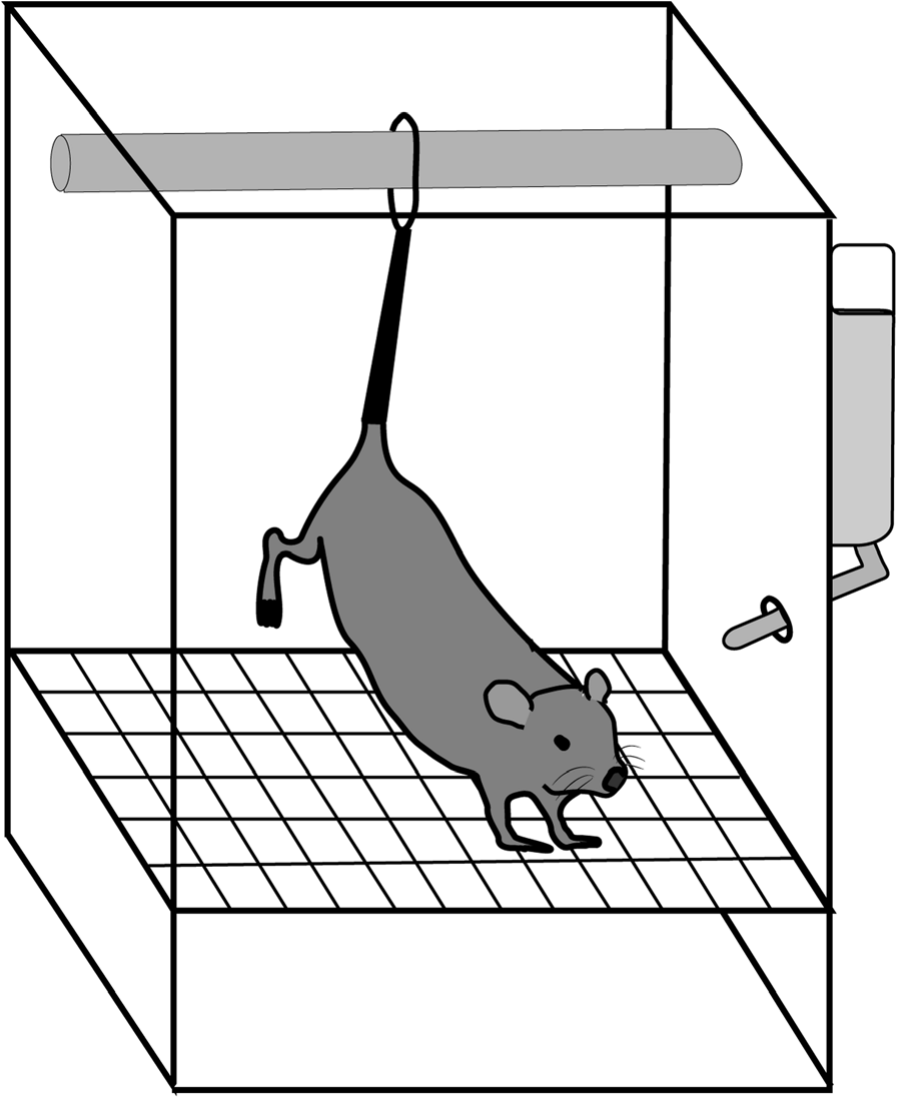
Tail suspended mouse model. A model in which the tail of a mouse is suspended to release the hind limbs from mechanical load

Twenty 12-week-old male C57BL6/NcL mice (*n* = 5/group) weighing about 25 g were used as experimental animals. The mice were divided into two groups: the experimental group (*n* = 10) which were reared by tail suspension, and the control group (*n* = 10) which were reared normally. The mice were first reared normally for 1 week before starting tail suspension. On the 21st day after tail suspension, an implant was placed into the left central portion of the femoral shaft of 5 animals in each group while the remaining 5 animals were sacrificed without implants, and the femurs were excised. The mice with implants were classified into a tail suspension group (TS.IP) and a normal rearing group (CTL.IP). The mice without implants were also classified into a tail suspension group (TS) and a normal rearing group (CTL). The healing period after implant placement was 3 weeks (Fig. 1). The method of tail suspension was according to the method of Holton et al. [1]. The tail of the mouse was disinfected, wrapped with elastic tape, a wire clip was connected, the wire was connected to the ceiling of the tail suspension cage (Fig. 2). The animal experiment was performed with the approval of the Tokyo Dental College Animal Experiment Committee (approval number 193303).

### Implant surgery

**Fig. 3:**
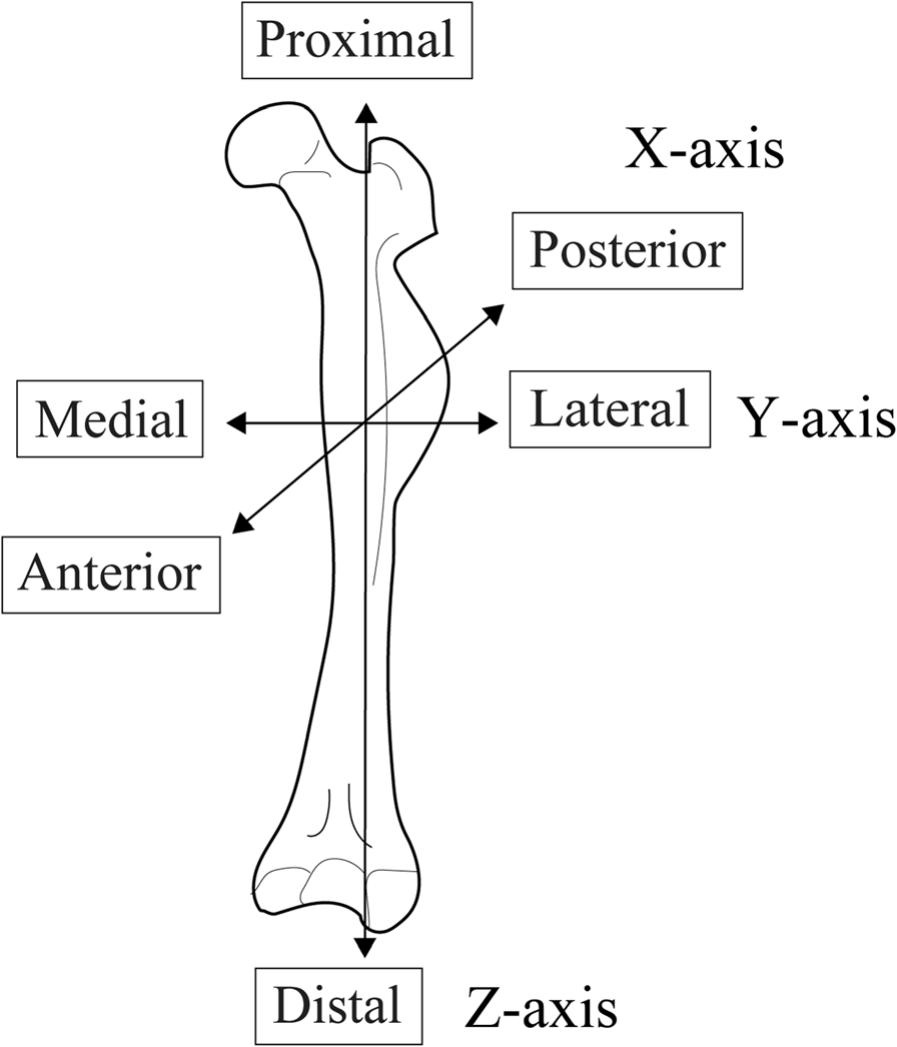
Femoral coordinate axis. The line connecting the lateral condyle with the greater trochanter of the femur was defined as the long axis of the femur, and the measurement directions of the three axes were set. *X* axis: anterior-posterior direction, *Y* axis: mesio-lateral direction, *Z* axis: femoral long-axis direction

**Fig. 4:**
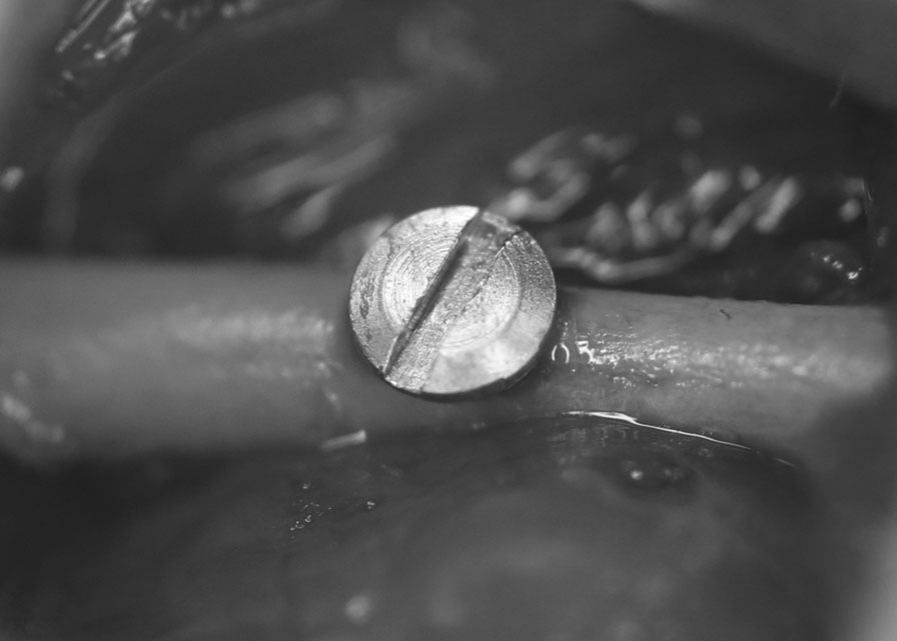
Implant placement surgery in the femur. A Ti-6Al-4V implant (length 1.0 mm, diameter 0.8 mm) was placed in the *Y*-axis direction in the center of the femoral shaft

The antero-posterior direction of the mouse femur was set as the *X*-axis, the medio-lateral direction as the *Y*-axis, and the long-axis direction as the *Z*-axis, as the reference axes of the sample (Fig. 3). Implant placement surgery was performed under general anesthesia by intraperitoneal administration of a combination of three anesthetics (medetomidine hydrochloride, 0.75 mg/kg, Nippon Zenyaku Kogyo Co., Ltd., Fukushima, Japan; midazolam, 4.0 mg/kg, Sand Co., Tokyo, Japan; butorfar tartrate, 5.0 mg/kg, Meiji Seika Pharma Stocks Co., Tokyo, Japan). From the ventral side of the mouse, approximately 20 mm of the skin just above the left femur was incised, the muscle layer was detached so as to clearly expose the femur. After peeling the periosteum, drilling was performed in the *Y*-axis direction using a standard drill (diameter 0.5 mm and 0.8 mm, Tamiya Co., Ltd., Shizuoka, Japan) under water injection in the center of the femoral shaft (10 mm above the knee joint). Next, a titanium alloy implant (Ti-6Al-4V, length 1.0 mm, diameter 0.8 mm, Nishimura Metal Co., Ltd., Fukui, Japan) was placed below 2 Ncm (Fig. 4). After that, the muscle layer and the skin were sutured with a 5-0 nylon thread (Mani Inc., Tochigi, Japan).

### Histological evaluation

**Fig. 5:**
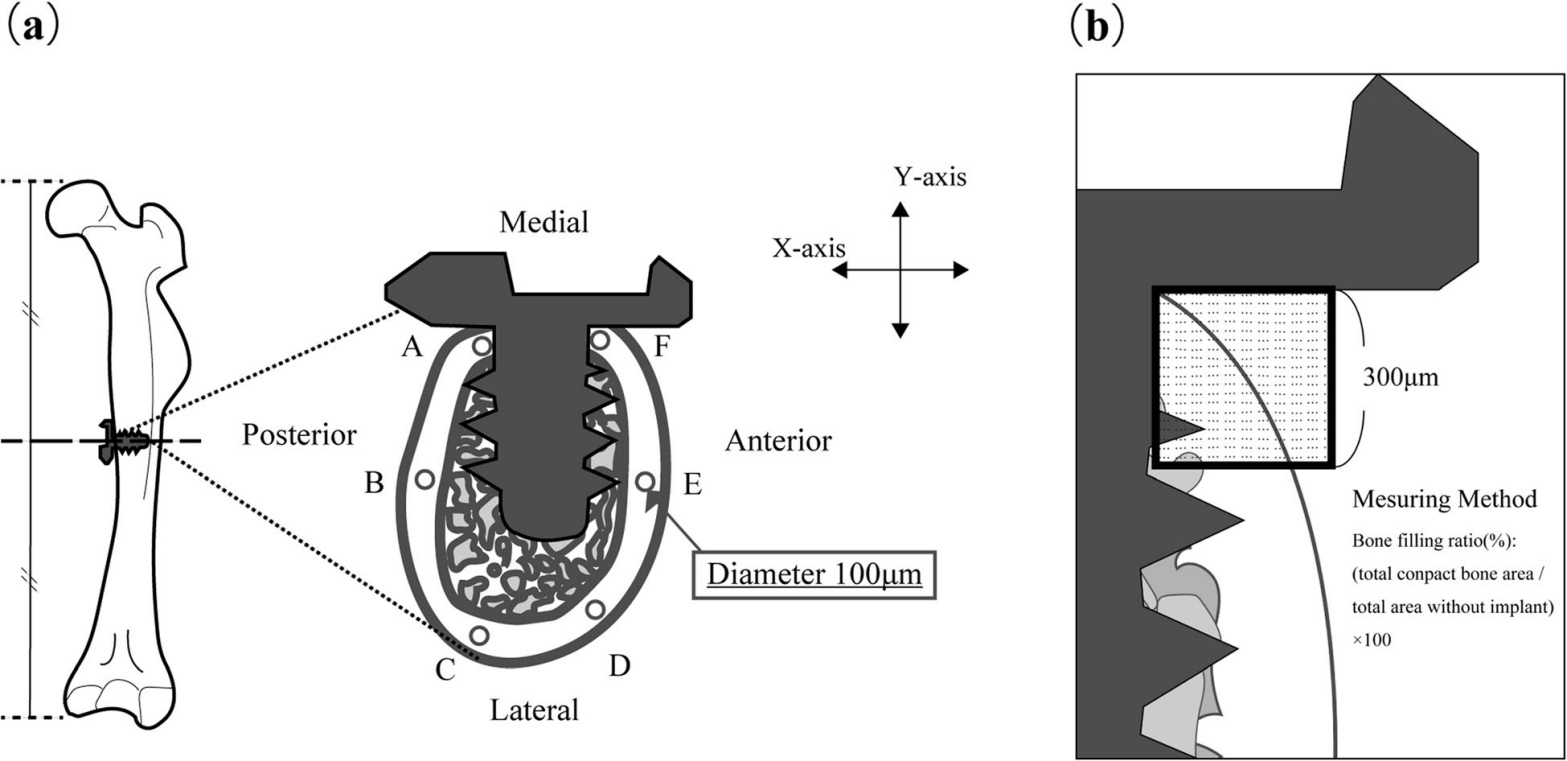
Method of setting region of interest and evaluating new bone mass surrounding the implant. **a** The region of interest was the cortical bone region on a 100 μm polished specimen created in the XY plane that passes through the center of the implant body. The measurement points were classified as follows: A, F: peri-implant area. B, C, D, E: normal area. **b** To evaluate the amount of new bone surrounding the implant, the value obtained by dividing the area occupied by calcified bone in the 300 μm square shown in the figure by the total area excluding the implant was shown as a percentage

All samples were immersed and fixed in 10% neutral buffered formalin for 2 days. Dehydration through graded ethanol and Villanueva osteochrome bone stain (Funakoshi Co., Ltd., Tokyo, Japan) were performed and cleared with styrene monomer (Nisshin EM, Tokyo, Japan). It was embedded in unsaturated polyester resin (Rigolac, Nissin EM, Tokyo, Japan) based on the *X-*, *Y-*, and *Z*-axes. The polymerized block was sliced in the XY plane passing through the center of the implant body using a saw microtome (SP1600, Leica, Wetzlar, Germany) with a blade width of 300 μm. Next, 100-μm-thick polished specimens were prepared with water-resistant abrasive paper (#400, #800, #1200). The sample obtained was observed with a universal optical microscope (Axiophot2, Carl Zeiss, Oberkochen, Germany). Using the attached image software (Axiovision, Carl Zeiss, Oberkochen, Germany), we measured the cortical bone thickness at 6 points A to F (Fig. 5a). For the measurement of new bone mass, we calculated the bone filling ratio of new bone within a 300 μm × 300 μm square at the implant neck (Fig. 5b).

### Morphometric evaluation of cancellous bone

The morphology of cancellous bone was evaluated using Micro-CT (μCT50; Scanco Medical AG, Brüttisellen, Switzerland) under the following conditions: tube voltage, 90 kV; tube current, 155 μA; matrix size, 3400 × 3400; slice width, 2 μm; and slice pitch, 2 μm. The region of interest was the cancellous bone region in the range of 1.6 mm proximo-distal from the center of the implant (the diameter of the implant body was 0.8 mm). To remove artifacts by implant, a copper plate filter of 0.5 mm was used for photography. In the three-dimensional structural analysis, the parameters of bone volume fraction (BV/TV; %), trabecular number (Tb.N; 1/mm), trabecular thickness (Tb.Th; μm), and trabecular separation (Tb.Sp; μm) were calculated.

### SHG imaging

Second harmonic generation (SHG) images were obtained using a multiphoton confocal microscopy system (LSM 880 Airy NLO, Carl Zeiss, Oberkochen, Germany) with excitation laser (Chameleon Vision II, wavelengths: 680–1080 nm; repetition rate: 80 MHz pulse width: 140 fs, Coherent Inc., Santa Clara, CA, USA) and objective lens (Plan-Apochromat 10x/0.8 M27, Carl Zeiss, Oberkochen, Germany). The excitation wavelength for observing collagen fibers was 880 nm. From the image obtained, a region of 200 μm × 200 μm square at the implant neck (A and F) was extracted as the region of interest. High-precision image analysis software (Imaris8.4, Bitplane AG, Zürich, Switzerland) was used to trace and measure the angles of the collagen fiber bundles.

### BAp crystal orientation

Quantitative evaluation of the BAp crystal orientation was performed using a curved imaging plate X-ray diffractometer (XRD, D/MAX RAPIDII-CMF, Rigaku Co., Ltd., Tokyo, Japan) of the optical system. Using an optical microscope attached to XRD (0.6–4.8x), the irradiation area was positioned at the center of the cortical bone, and X-rays were irradiated so that the incident beam became a circle with a diameter of 100 μm. The position of the sample was determined according to the *X*-axis, *Y*-axis, and *Z*-axis as the reference axes. At the implant neck, the location was 50 μm away from the implant surface in the *X*-axis direction. The measurement was performed at 6 points A to F (Fig. 5a) of a 100-μm-thick specimen. The points of intersection of a line drawn from the midpoint of the femur width in the *Y*-axis direction parallel to the *X*-axis and the cortical bone were designated as B and E, and C and D were set from A and F on a vertical line in the *Y*-axis direction. As according to Nakano et al. [18], the measurement was performed by two methods, a transmission type optical system and a reflection type optical system, and Cu-Kα rays were used as the radiation source. The setting conditions were a tube voltage of 40 kV and a tube current of 30 mA.

From the diffraction ring image drawn on the imaging plate with the diffracted X-ray beam, the X-ray intensity ratio of the two diffraction peaks of the (002) plane and the (310) plane was calculated using 2D Date Processing Software (Rigaku Inc., Tokyo, Japan).

### Statistical analysis

Statistical analysis was performed using GraphPad Prism version 6.0 (GraphPad Software Inc., San Diego, CA, USA). After each measurement, one-way analysis of variance and Tukey’s multiple comparison test were performed. In addition, a *t* test was used to compare the new bone mass. A statistically significant *p*-value was less than 0.05.

## Results

### Histological evaluation

**Fig. 6:**
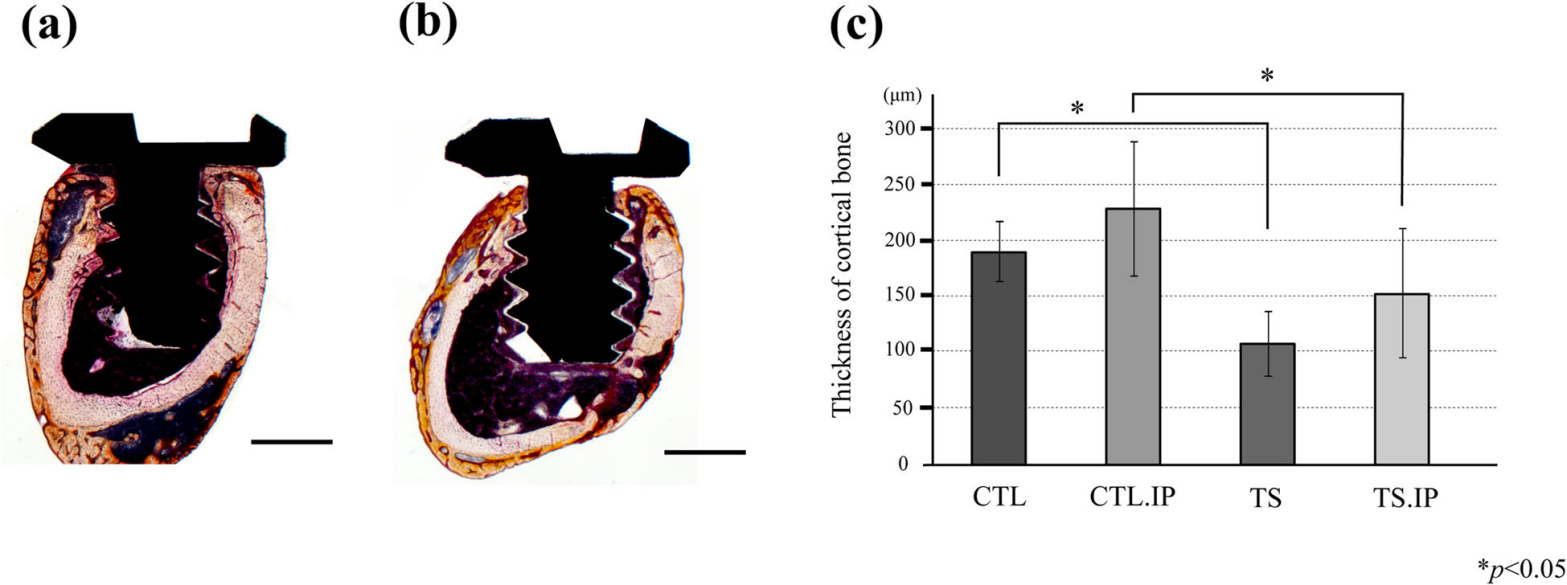
Comparison of cortical bone thickness in each group. Villanueva bone stained image. **a** CTL.IP. 25× magnification. **b** TS.IP. 25× magnification. **c** Horizontal axis shows groups, vertical axis shows cortical bone thickness (μm). The thickness of cortical bone was significantly higher in the CTL and CTL.IP than in the TS and TS.IP. Bar: 500 μm

**Fig. 7:**
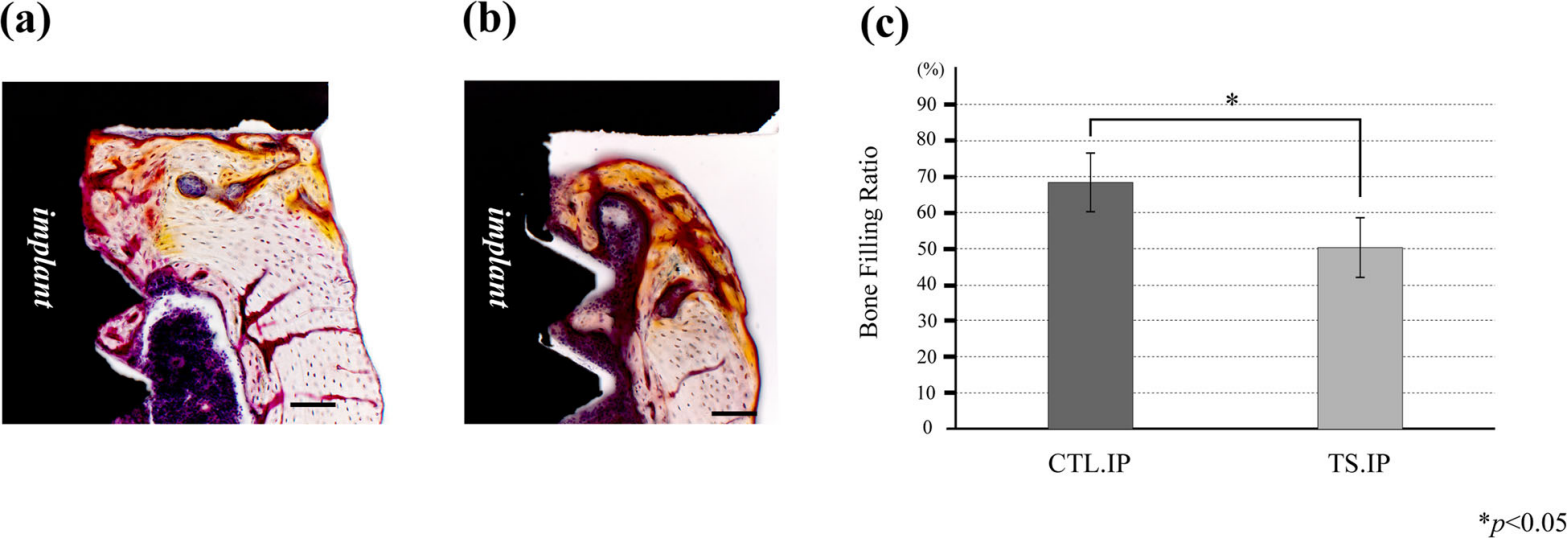
Quantitative evaluation by the bone filling ratio surrounding the implant. **a** CTL.IP. Peri-implant area. 100× magnification. **b** TS.IP. Peri-implant area. 100× magnification. **c** Comparison of bone filling ratio. The horizontal axis shows the groups, and the vertical axis shows the bone filling ratio (%). Bar: 100 μm

In both groups, lamellar bone was observed surrounding the implant (Fig. 6a, b). The thickness of cortical bone at each site (A–F) was significantly lower in the tail suspension groups (TS, TS.IP) (Fig. 6c). In the implant group, new bone showing a cortical bone-like structure was observed surrounding the implant in all samples (Fig. 7a, b). The bone filling ratio of new bone at the implant neck (A, F) was significantly higher in the CTL.IP than in the TS.IP (Fig. 7c).

### Morphometric evaluation of cancellous bone

**Fig. 8:**
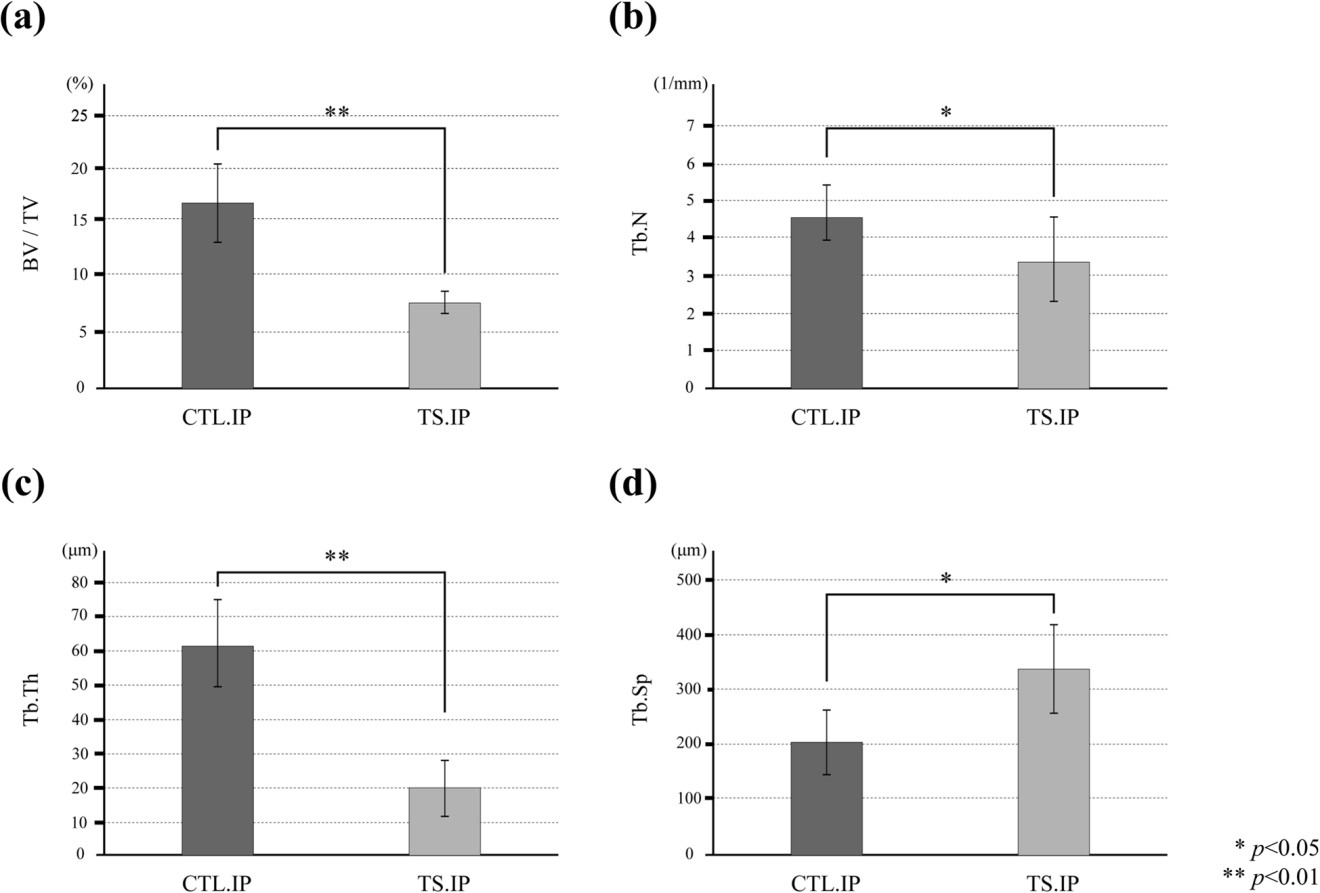
Morphometric evaluation of cancellous bone. **a** BV/TV. **b** Tb.N. **c** Tb.Th. **d** Tb.Sp

The morphometric parameters BV/TV, Tb.N, and Tb.Th in the TS.IP were significantly lower than those of the CTL.IP (Fig. 8a–c). And Tb.Sp in the TS.IP was significantly higher than in the CTL.IP (Fig. 8d).

### SHG imaging

**Fig. 9:**
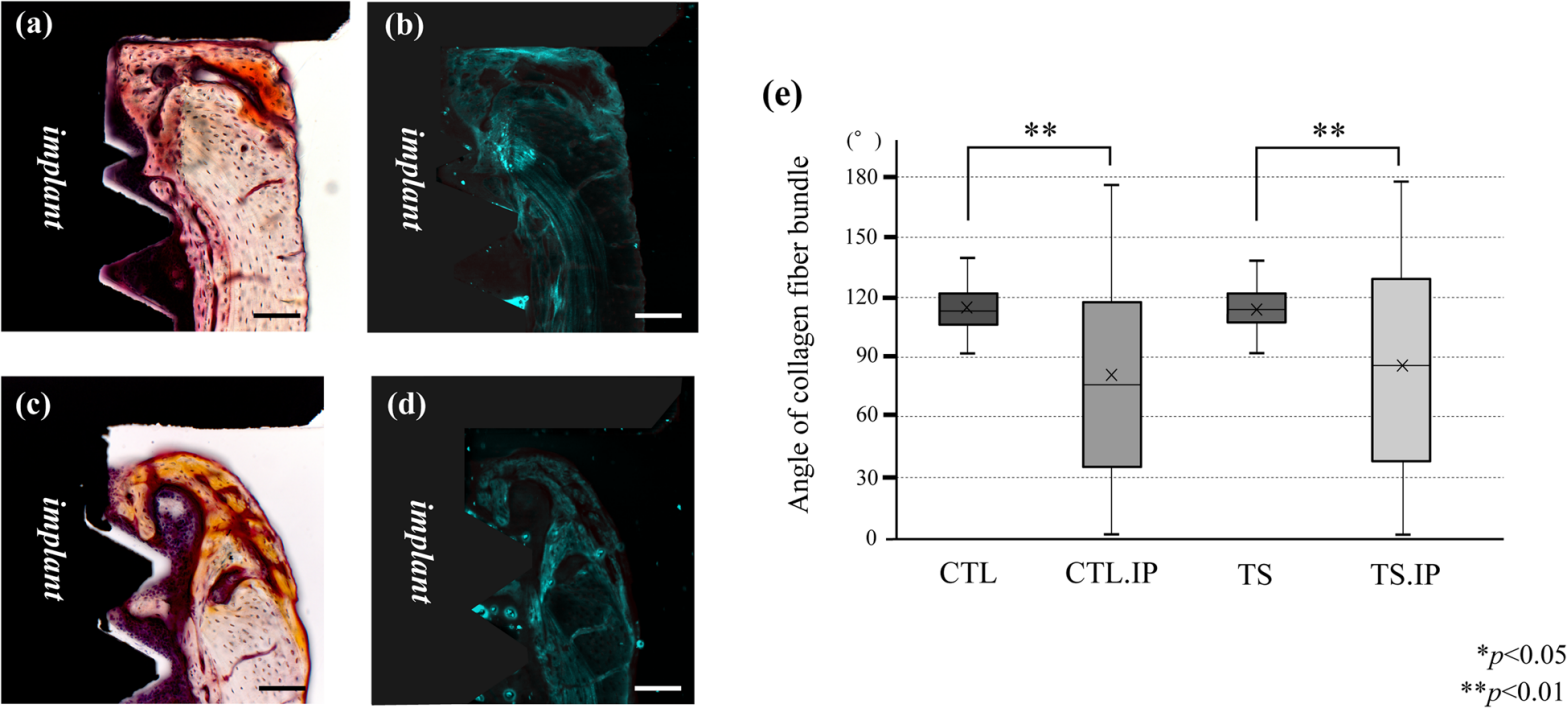
SHG imaging in the peri-implant area. **a** Optical microscope image of CTL.IP. **b** SHG imaging of CTL.IP. **c** Optical microscope image of the TS.IP. **d** SHG imaging of the TS.IP. **a**–**d** 100× magnification. Bar: 100 μm. **e** Box-and-whisker plot of the angle of the collagen fiber bundle with respect to the *Y*-axis

SHG imaging confirmed collagen fiber bundles with distinctly different running in the peri-implant bone and normal bone. Collagen fiber bundles in the bone surrounding the implant tended to run in random directions, and in normal bone they tended to run parallel to the endoperiosteum (Fig. 9a–d). Collagen fiber bundles were often observed near the endoperiosteum at a site (B–E) sufficiently far from the implant neck, which is considered to be normal bone. The running of collagen fibers surrounding the implant was extremely irregular (mean ± SD), and was 81.1° (± 50.6) in the CTL.IP and 85.6° (± 52.2) in the TS.IP with respect to the *Y*-axis. At the same site (A and F) in the non-implant group, the CTL was 114.9° (± 11.5) and the TS was 113.7° (± 10.8) with respect to the *Y*-axis (Fig. 9e). The running of collagen fiber bundles was irregular in the implant groups (CTL.IP, TS.IP) compared to the non-implant groups (CTL, TS).

### BAp crystal orientation

**Fig. 10:**
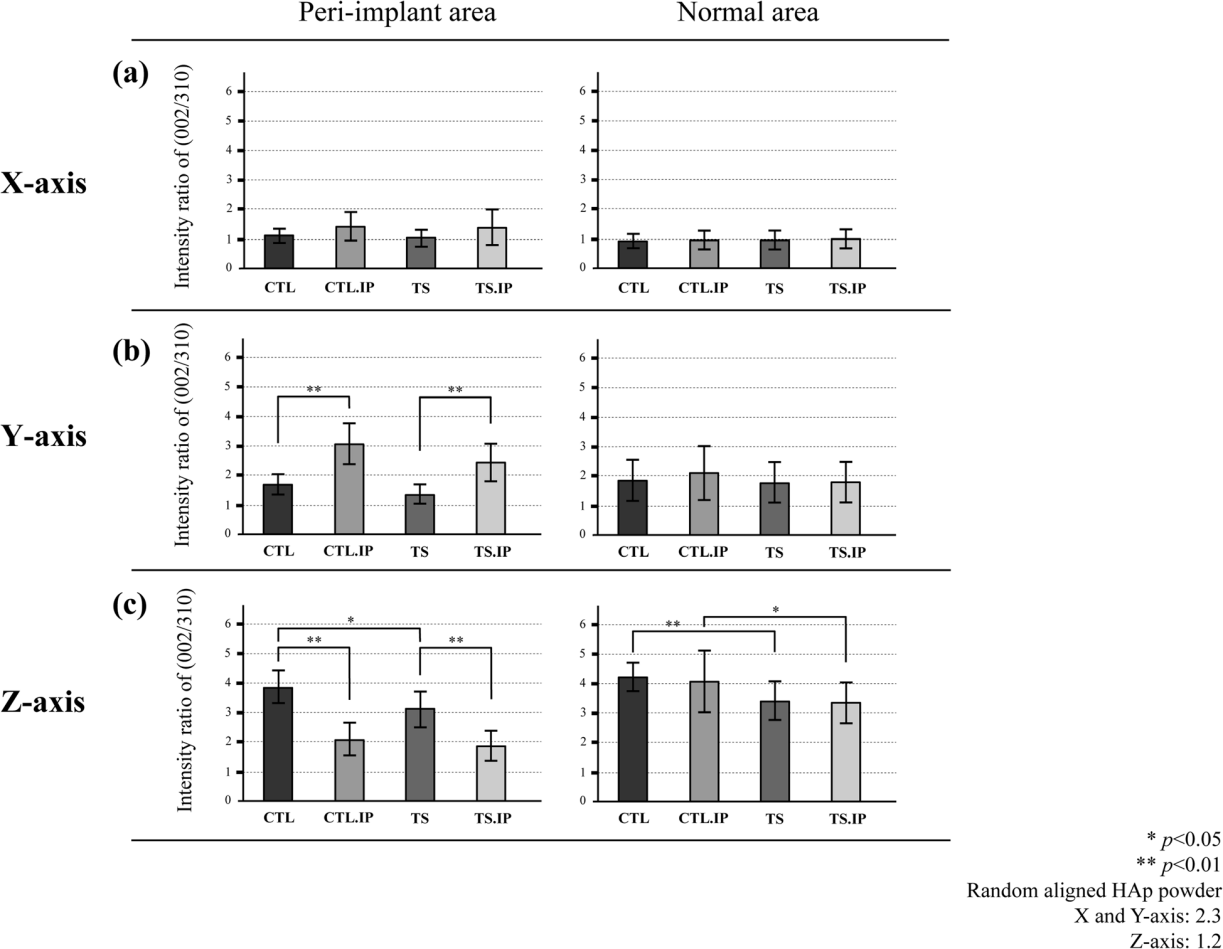
Orientation of BAp c-axis in each measurement axis. The vertical axis shows the X-ray diffraction intensity ratio calculated from the (002)/(310) peaks, and the horizontal axis shows the groups. **a** *X*-axis (antero-posterior) direction. **b** *Y*-axis (mesio-lateral) direction. **c** *Z*-axis (femoral long-axis) direction

In all groups (CTL, CTL.IP, TS, TS.IP), no preferential orientation of BAp *c*-axis in the *X*-axis direction was observed (Fig. 10a). In the peri-implants area (A, F), no preferential orientation of BAp *c*-axis in the *Y*-axis direction was observed in the non-implant groups (CTL, TS), but preferential orientation of BAp *c*-axis in the *Y*-axis direction were observed in the implant groups (CTL.IP, TS.IP) (Fig. 10b). No difference in the X-ray diffraction intensity ratio in the *Y*-axis direction was observed in the normal bone areas (B, C, D, E). In the non-implant groups (CTL, TS), preferential orientation of BAp *c*-axis was observed in the *Z*-axis direction at all sites (A–F). In the normal rearing groups (CTL, CTL.IP), the X-ray diffraction intensity ratio in the *Z*-axis showed a higher value than in the tail suspension groups (TS, TS.IP). At the implant neck (A, F) in the implant groups (CTL.IP, TS.IP), preferential orientation of BAp *c*-axis in the *Z*-axis were existed. However, the X-ray diffraction intensity ratio in the *Z*-axis in the implant groups (CTL.IP, TS.IP) were significantly lower than that in the non-implant groups (CTL, TS) (Fig. 10c).

## Discussion

From histological observation, the newly formed bone tissue surrounding the implant had a lamellar bone regardless of the loading conditions. This result was mostly consistent with the shape of the bone surrounding the implant reported by Villar et al. [18]. Similarly to the previous report [19], cortical bone and bone marrow tissue were in contact with the implant at the interface between the implant and bone, and inclusion of bone cells and blood vessels in the cortical bone was observed. On the other hand, in the TS.IP, it was found that the cortical bone thickness and the bone mass measured surrounding the implant showed low values. Frost [20] reported that the increase and decrease of bone mass changed in response to microstrain, and that the strain above a certain level is required to maintain or renew bone mass. In addition, Degidi et al. [21] reported that the occlusal force applied to the bone through the implant contributes to the increase in bone mass surrounding the implant. In this study, in which the load applied to the bone surrounding the implant was reduced as much as possible, the thickness of cortical bone and the increment of new bone were small, but showed the same tendency as normal rearing groups (CTL, CTL.IP). This suggests that osseointegration occurs at the implant body interface after implant placement even under low load conditions.

As a result of analysis by SHG imaging, collagen fiber bundles were found running almost parallel to the endoperiosteum in normal mouse femurs, whereas collagen fiber bundles running in new bone surrounding the implant were very irregular. It was suggested that the collagen fiber bundles were rearranged into an irregular shape by implant placement. However, no significant difference was observed between the implant groups (CTL.IP, TS.IP). This is considered to be because the wound healing process due to implant placement had a far greater effect than loading conditions.

On the other hand, regarding BAp crystal orientation, Nakajima et al. [22] reported that the uniaxial preferential orientation of BAp *c*-axis in the longitudinal direction of the femur was diminished by tail suspension rearing for 3 weeks in mice, but the minimum uniaxial preferential orientation for maintaining the bone structure remained. In the non-implant groups (CTL, TS), a uniaxial preferential orientation of BAp *c*-axis was observed in the *Z*-axis direction. But, in the TS, the X-ray diffraction intensity ratio in the *Z*-axis direction was significantly lower than that in the CTL. These results were mostly consistent with the previous reports [22, 23]. Therefore, it seems that the femur adapted to a new loading environment after it lost homeostasis due to changes in the mechanical environment.

Otherwise, at the bone surrounding the implant, the X-ray diffraction intensity ratio in the *Z*-axis direction in the implant groups (CTL.IP, TS.IP) showed lower value than in the non-implant groups (CTL, TS). On the other hand, the X-ray diffraction intensity ratio in the *Y*-axis direction was high value after implant placement. From the results of the orientation in the *Y*-axis direction observed in the implant groups (CTL.IP, TS.IP), it is inferred that the residual stress during implant placement mainly occurred in the *Y*-axis direction [24, 25]. On the other hand, no significant difference was observed in the X-ray diffraction intensity ratio in the Y-axis direction in the implant groups (CTL.IP, TS.IP), thus similar to collagen fiber bundles, implant placement is considered to have a much larger effect than the unloading associated with tail suspension. In addition, Odaka et al. [26] reported in animal experiments using beagle dogs that the preferential orientation of BAp *c*-axis in the bone surrounding the implant changed in the direction of occlusal force.

Due to the tail suspension rearing, the femur of the mouse receives almost no functional pressure from the muscle in addition to its own weight. In the oral cavity, this state imitates the alveolar bone excluding loads such as occlusal force and muscle function pressure. In other words, this model was created assuming that the implant was placed in the jawbone, which was hardly affected by the load for a long time after tooth extraction. The results of this study showed that osseointegration and surrounding bone renewal that occur immediately after implant placement can be obtained with almost no load. However, since the bone mass and quality were significantly lower than those in the control group, it was suggested that various functional pressures generated in the oral cavity are required to obtain good osseointegration and peri-implant bone at the initial stage of implant placement.

## Conclusions

As a result, it was revealed that even under extremely low load conditions, bone formation occurs surrounding the implant, and bone microstructure and bone quality adapted to the new mechanical environment are obtained. Clinically, it was suggested that implant placement creates new bone structural characteristics surrounding the implant.

## Data Availability

The datasets used and analyzed during the current study are available from the corresponding author on reasonable request.

## Abbreviations

BAp: Biological apatite
CTL: Control
CTL.IP: Control with implant
TS: Tail suspension
TS.IP: Tail suspension with implant
XRD: X-ray diffraction
SHG: Second harmonic generations
